# Breakthroughs in Medicinal Chemistry: New Targets and Mechanisms, New Drugs, New Hopes

**DOI:** 10.3390/molecules22050743

**Published:** 2017-05-05

**Authors:** Diego Muñoz-Torrero, Arduino A. Mangoni, Catherine Guillou, Simona Collina, Jean Jacques Vanden Eynde, Jarkko Rautio, György M. Keserű, Christopher Hulme, Kelly Chibale, F. Javier Luque, Rafik Karaman, Michael Gütschow, Hong Liu, Rino Ragno

**Affiliations:** 1 Laboratory of Pharmaceutical Chemistry, Faculty of Pharmacy and Food Sciences, and Institute of Biomedicine (IBUB), University of Barcelona, Av. Joan XXIII; 27-31, E-08028 Barcelona, Spain; 2 Department of Clinical Pharmacology, Flinders University and Flinders Medical Centre, Bedford Park, SA 5042, Australia; arduino.mangoni@flinders.edu.au; 3 Institut de Chimie des Substances Naturelles, CNRS UPR 2301, Université de Paris-Saclay, 91198 Gif-sur-Yvette, France; catherine.guillou@cnrs.fr; 4 Department of Drug Sciences, Medicinal Chemistry and Pharmaceutical Technology Section, Centre for Health Technologies (CHT), University of Pavia, Viale Taramelli 12, 27100 Pavia, Italy; simona.collina@unipv.it; 5 Organic Chemistry Laboratory, University of Mons-UMons, 7000 Mons, Belgium; Jean-Jacques.VANDENEYNDE@ex.umons.ac.be; 6 School of Pharmacy, Faculty of Health Sciences, University of Eastern Finland, P.O.Box 1627, FI-70211 Kuopio, Finland; jarkko.rautio@uef.fi; 7 Medicinal Chemistry Research Group, Research Centre for Natural Sciences, The Hungarian Academy of Sciences, Magyar tudósok körútja 2, H-1117 Budapest, Hungary; keseru.gyorgy@ttk.mta.hu; 8 Department of Pharmacology and Toxicology, College of Pharmacy, The University of Arizona, Tucson, AZ 85721, USA; hulme@pharmacy.arizona.edu; 9 Department of Chemistry, Institute of Infectious Disease and Molecular Medicine, and South African Medical Research Council Drug Discovery and Development Research Unit, University of Cape Town, Rondebosch 7701, South Africa; kelly.chibale@uct.ac.za; 10 Department of Nutrition, Food Science, and Gastronomy and Institute of Biomedicine, University of Barcelona, Av. Prat de la Riba 171, 08921 Santa Coloma de Gramenet, Spain; fjluque@ub.edu; 11 Pharmaceutical & Medicinal Chemistry Department, Faculty of Pharmacy, Al-Quds University, POB 20002 Jerusalem, Palestine; dr_karaman@yahoo.com; 12 Department of Sciences, University of Basilicata, Viadell’Ateneo Lucano 10, 85100 Potenza, Italy; 13 Pharmaceutical Institute, University of Bonn, An der Immenburg 4, 53115 Bonn, Germany; guetschow@uni-bonn.de; 14 Key Laboratory of Receptor Research, Shanghai Institute of Materia Medica, Chinese Academy of Sciences, 555 Zu Chong Zhi Road, Shanghai 201203, China; hliu@simm.ac.cn; 15 Rome Center for Molecular Design, Department of Drug Chemistry and Technology, Sapienza University, P.le Aldo Moro 5, 00185 Rome, Italy; rino.ragno@uniroma1.it

## 1. Introduction

The Editorial Board of the Medicinal Chemistry section of the journal *Molecules* publishes here its first *Editorial*, which has been prepared by highlighting, in sub-editorials of about one hundred words, some selected recently published articles that may have a profound impact on drug discovery and therapy.

In particular, this editorial highlights new drug targets and mechanisms of action and new classes of drugs, as well as new therapeutic uses for known drugs or the involvement of known biological targets in new diseases. We also discuss some structural biology studies and new computational tools that may pave the way for the rational design or identification of more efficacious and safer drugs. Overall, the findings reported in these highlighted papers raise our hopes for the management of difficult-to-treat diseases that are posing a growing health threat, with new or repurposed drugs that overcome the limitations of currently applied therapies.

## 2. Hitting the Protozoan Parasite Proteasome: A New Avenue for Antimalarial and Antitrypanosomatid Drug Discovery

### Highlight by Diego Muñoz-Torrero

Protozoan diseases cause immense human suffering and death. Available drugs have a number of drawbacks that make necessary the search for alternative antiprotozoal agents that hit novel parasite targets. Two independent reports have shown that proteasome inhibition can derive potent and selective compounds against major protozoan diseases. On the basis of the specificity profiles of *Plasmodium falciparum* and human proteasomes and structural studies, Li et al. rationally designed a peptidic inhibitor of the parasite proteasome, which has shown efficacy and good tolerability in a *Plasmodium* infection mouse model [1]. After the phenotypic screen of 3 million compounds against *Leishmania donovani*, *Trypanosoma cruzi*, and *Trypanosoma brucei* and hit-to-lead optimization, Khare et al. identified (i) a lead compound that has shown compelling efficacy in mouse models of visceral and cutaneous leishmaniasis, Chagas disease, and human African trypanosomiasis and (ii) the parasites’ proteasome as the pan-kinetoplastid target of this class of compounds [2]. Let’s keep our fingers crossed that proteasome inhibitors will be soon combating malaria and trypanosomatid diseases!

## 3. Sulfonamidoacetamides: A Novel Treatment Strategy for Axon Regeneration?

### Highlight by Arduino A. Mangoni

New drugs that effectively stimulate axon growth are urgently required as an increasing number of patients suffer from the sequelae of neurological insults. After screening the chemical libraries of ~170,000 synthetic small molecules, Ku et al. identified a set of compounds with a common chemical scaffold containing a core substructure of sulfonamidoacetamide, stimulating neurite outgrowth in P19 embryonic carcinoma cells and rat primary hippocampal neurons [3]. Structure-activity guided optimization resulted in the identification of compound 2-(*N*-(2-methoxyphenyl)-4-methylphenylsulfonamido)-*N*-(4-methoxypyridin-3-yl)acetamide, characterized by relatively high metabolic stability and significant effects on axon growth both in retinal neuronal cells and in an animal model of optic nerve injury [3]. Pending further pharmacodynamic/pharmacokinetic characterization and optimization, this study might pave the way for the development of a new class of drugs stimulating axon regeneration.

## 4. New Hope against Growing Threat of Antibiotic Resistance

### Highlight by Catherine Guillou

The modulation of bacterial communication (Quorum Sensing, QS) to potentiate the effect of existing antimicrobial drugs is a promising alternative to the development of new antibiotics. *Staphylococcus aureus* is an important causative agent of acute and chronic bacterial infections in humans as well as in animals. In addition, *S. aureus* often reside within the biofilm at the site of infection, displaying enhanced resistance to antibiotics. Hamamelitannin (HAM), a natural product, was shown to potentiate the effects of antimicrobials against *S. aureus* by interfering with QS. HAM affects *S. aureus* biofilm susceptibility through the trapP receptor by affecting cell wall synthesis and extracellular DNA release of *S. aureus*. Furthermore, this compound increases the susceptibility of *S. aureus* biofilm towards different classes of antibiotics in vitro and in vivo [4]. An analogue of HAM was found to be more potent than HAM [5].

## 5. Targeting the Protein-RNA Complexes: A New Opportunity for Medicinal Chemists

### Highlight by Simona Collina

RNA-protein interactions are of pivotal importance in the regulation of biological systems and are implicated in gene expression defects. RNAs in cells are in complex with proteins called RNA-binding proteins (RBPs), which influence every aspect of the biogenesis and function of RNAs. In their recent paper, Hall and coworkers showed the potential of the complex formed by Lin28 (RBP) and let-7 (RNA), as a new target for selected cancers; they furthermore discovered an antagonist of Lin28. Much can be learned from this report, including the drug target potential of other RBP:RNA complexes involved in pathophysiological mechanisms. This pioneering paper suggests a fascinating route for discovering new drugs able to inhibit or enhance gene expression [6].

## 6. Repurposing Pentamidine: A New Hope for the Treatment of Gram-Negative Infections

### Highlight by Jean Jacques Vanden Eynde

Pentamidine, 4,4’-[1,5-pentanediylbis(oxy)]dibenzenecarboximidamide, is a bisbenzamidine in clinical use for the treatment of Human African trypanosomiasis, visceral leishmaniasis, and *Pneumocystis jiroveci* pneumonia. Its activity is typically correlated to a strong affinity for the DNA minor groove, but other intracellular targets have also been discovered [7]. The research group of E.D. Brown [8] established that pentamidine was able to perturb the outer membrane of Gram-negative pathogens through association with lipopolysaccharides and, in this case, without internal interaction. Starting from that result, the study demonstrated that the diamidine could effectively act, in vitro, as a potent adjuvant to enhance the sensitivity of polymyxin-resistant bacteria to Gram-positive antibiotics such as rifampicin, erythromycin, and novobiocin. In vivo, a promising dose-sparing effect has been observed when a combination of pentamidine and novobiocin was used for the treatment of systemic *Acinetobacter baumannii* infections in mice. A novel potential use for pentamidine!

## 7. Fragment-Based Screening in Human Cells

### Highlighted by Jarkko Rautio

A collaborative effort from the Scripps Research Institute, the University of Lausanne, Bristol-Myers Squibb and the Salk Institute ingeniously demonstrates a chemical proteomic platform to map small-molecule fragment-protein interactions directly in human cells. Typically, fragment-based ligand and drug discovery (FBLD) emphasizes the identification of structurally simple hit compounds by screening ~10^3^ low-molecular weight compounds (<300 Da) at high concentrations (>100 µM) in in vitro assays only with purified proteins. The group used the photoaffinity/click-label combination to discover more than 2000 fragment–binding proteins in HEK293T cells, only a few of which are found in DrugBank database. Using the quantitative MS-based SILAC (stable isotope labeling with amino acids in cell culture) technique, they quantified and identified labeled proteins and tracked down poorly characterized transmembrane protein *PGRMC2* (progesterone receptor membrane component 2) for which selective ligands were previously lacking. This is an excellent article for those into early-stage drug discovery and medicinal chemistry in general, and it likely paves the way to a huge amount of useful research in the future [9].

## 8. The Atomic Level Mechanism of LSD Action Has Been Solved

### Highlight by György M. Keserű

The research group of Bryan L. Roth at the University of North Carolina, USA published the high-resolution X-ray structure of Lysergic acid diethylamide (LSD) bound to serotonin 2B receptor, one of its in-cell brain targets [10]. This study revealed for the first time how LSD is captured by its receptor and described the conformational change at the binding site that closes the drug to the pocket like a lid. This binding mechanism was connected to the long residence time of LSD that is responsible for its long duration of action. This kind of interaction seems to be specific for LSD, since other compounds such as the vasoconstrictor drug ergotamine might leave the binding site of the serotonin 2B receptor more open [11]. Structural data also help elucidate the activation of the receptor. It has been demonstrated that LSD activates both the G-protein dependent and the G-protein independent β-arrestin signalling pathways. Interestingly the team found that increasing the mobility of the lid by introducing mutation L209A not only facilitated the unbinding of the drug, but more importantly decreased β-arrestin2 recruitment significantly. Considering the option for functionally selective drugs with preference for specific signalling pathways, the new crystal structure of the LSD-serotonin 2B receptor complex could help in designing safer psychiatric drugs with less side effects.

## 9. PIM-1 Inhibition as a Novel Therapeutic Strategy for Alzheimer’s Disease

### Highlighted by Christopher Hulme

Targeting Central Nervous System (CNS) kinases with small molecule inhibitors to deliver therapeutics for neurodegeneration has so far proved elusive. An article by Oddo et al. [12] revealed new utility for PIM-1 kinase, outside the oncology realm, to address Alzheimer’s disease. A selective brain penetrable PIM-1 inhibitor, (3-cyano-4-phenyl-6-(3-bromo-6-hydroxy)phenyl-2(1*H*)-pyridone), was evaluated in 3xTg-AD mice, demonstrating significant reductions in both Aβ and tau pathology with improvements in cognitive deficits. At the heart of this effect is a proposed reduction of mTOR activity, mediated by the inhibition of PIM-1-driven PRAS40 phosphorylation. With several PIM-1 inhibitors in trials, opportunities to rapidly realign efforts toward AD can now be exploited.

## 10. Tubulin-Bound Conformation of a Hypermodified Epothilone Analog

### Highlight by Kelly Chibale

One approach to natural product-based medicinal chemistry is the development of synthetic technologies to facilitate the synthesis of new analogues for structure-activity relationship (SAR) studies. Karl-Heinz Altmann and his team [13] used ring-closing olefin metathesis as a key synthetic technology to prepare 12-aza-epothilones (also known as azathilones). Highly potent inhibitors of cancer cell growth in vitro were obtained, and meaningful SARs were delineated**.** Furthermore, the authors determined the conformation of a representative azathilone bound to the target tubulin heterodimers, which was found to be similar to that of the natural epothilones A and B. Although the bound conformation did not provide a molecular basis for the observed SAR, it provided an opportunity for further investigations aimed at gaining insight into target binding for this class of epothilone analogues.

## 11. Molecular Dynamics Simulations and Kinetic Measurements to Estimate and Predict Protein-Ligand Residence Time

### Highlighted by F. Javier Luque

The concept of residence time is gaining increasing impact in drug discovery, because the lifetime of drug occupancy is a key property for its pharmacological activity. However, its computation poses a serious challenge due to the high demands in computer time required for the sampling of drug unbinding, thus limiting practical industrial applications. In an effort to solve this question, Mollica et al. [14] reported an efficient computational approach that relies on simulating the target-ligand unbinding by using molecular dynamics under scaled potential energy conditions. This strategy facilitates the rupture of key intermolecular interactions, leading to unbinding events in much shorter simulation times. After a fine-tuning of the computational protocol, the results obtained for a series of glucokinase activators demonstrated that the calculated residence times were in agreement with the experimental data. Albeit extension to a larger number of target-ligand complexes is needed, the results support the potential of this technique for drug binding kinetics studies.

## 12. Natural Products as Sources of New Drugs from 1981 to 2014

### Highlight by Rafik Karaman

In this study, David J. Newman’s group comprehensively updated and broadened the data on the sources of new drugs which have been published in four different reviews during 1997–2012 [15]. Based on the new data covering the 34 years from 1 January 1981 to 31 December 2014, the group has concluded that natural products are the stepping stone in the discovery and development of drugs for the treatment of human diseases such as cancer, infectious (viral, microbial, and parasitic), antidiabetic, inflammation and hypertension treatments. Furthermore, the data demonstrated that the ideal solution to the current huge crisis facing the drug discovery and development sector can be achieved by a multidisciplinary approach, consisting of both novel molecular diversity generated from natural product sources and combinatorial synthetic procedures that rely on the manipulation of biosynthetic routes.

## 13. Proteasome Subunit Selective Activity-Based Probes

### Highlight by Michael Gütschow

Fifteen different types of core particles can exist in cells expressing all subunits of the constitutive (cCP) and immunoproteasome (iCP). To study the proteasome assemblies, Herman S. Overkleeft’s group at the Leiden Institute of Chemistry has designed and employed a panel of subtype selective, peptidomimetic epoxyketone- or vinyl sulfone-based probes equipped with either BOPIPY(FL) or Cy5 fluorophores as FRET pairs and targeting the caspase-, trypsin- or chymotrypsin-like activities of proteasomes [16]. Cell extracts were treated with purposeful combinations of these FRET donor/acceptor activity-based probes (ABPs) and resolved on native-PAGE. The intraproteasomal FRET was analyzed by in-gel fluorescence measurements. For example, when a donor ABP is bound to a cCP subunit and an acceptor ABP to an iCP subunit, or vice versa, which are assembled in the same core particle, mixed proteasomes can be detected. Asymmetric mixed proteasomes were analytically accessible as well. Moreover, the authors identified the predominantly produced proteasome types in HeLa cells, exposed to interferon-γ. Thus, a novel, sensitive and straightforward assay to identify proteasome core particle composition was introduced.

## 14. An Effective CADD Method for the Discovery and Rational Design of Natural Product Derivatives

### Highlighted by Hong Liu

DPP-4 inhibitors are considered a promising class of antidiabetic drugs. In particular, long-acting DPP-4 inhibitors are clinically needed for improving patients’ compliance with a safe, once daily or weekly dosing regimen. Li et al. [17] identified the natural product isodaphnetin to be a potent DPP-4 inhibitor (IC_50_ = 14 μM) through target fishing dock (TarFisDock). Utilizing highly efficient 3D molecular similarity-based scaffold hopping as well as electrostatic complementary methods, Li et al. discovered a series of novel 2-phenyl-3,4-dihydro-2*H*-benzo[*f*]-chromen-3-amine derivatives with highly potent DPP-4 inhibitory activities. The most potent inhibitor of this series exhibited an IC_50_ value of 2.0 nM, an approximately 7400-fold improvement of potency compared with isodaphnetin, which also displayed good oral bioavailability (*F* = 89%). In addition, this compound presented over 80% inhibition of DPP-4 at 3 mg/kg oral dose in 24 h, which is better than that of the long-acting positive control, omarigliptin. Moreover, this compound also improved the glucose tolerance at a comparable level with omarigliptin. In this study, an effective CADD method (TarFisDock) and medicinal chemistry strategies were successfully implemented to discover a promising DPP-4 inhibitor from a natural product, with therapeutic potential as a long acting antidiabetic agent [17].

## 15. New 1,4-Dihydropyridines as Sirt1 Activators as Potential Treatment for Dysmetabolic Syndrome

### Highlighted by Rino Ragno

Recently, Valente et al. [18] disclosed a series of 1,4-dihydropyridines (DHPs) displaying SIRT1 activation capability and high nitric oxide release in HaCat cells, which ameliorated skin repair in a mouse model of wound healing. In addition, DHPs derivatives showed to improve mitochondrial activity. The observed biological effects were impaired by co-administration of the AMPK inhibitor compound C or of the SIRT1 inhibitor EX-527, proving that the DHPs mechanism of action involves the SIRT1/AMPK pathway. Moreover, when tested in several cancer cell lines, water soluble DHP compounds displayed antiproliferative effects and increased H4K16 histone deacetylation. These findings will be of great utility to elucidate SIRT1 biological functions, such as its role in dysmetabolic syndrome and cancer.
